# Nursing care management concepts: scoping review

**DOI:** 10.1590/0034-7167-2022-0020

**Published:** 2023-02-06

**Authors:** Aurilívia Carolinne Lima Barros, Jouhanna do Carmo Menegaz, José Luís Guedes dos Santos, Sandra Helena Isse Polaro, Letícia de Lima Trindade, William Campo Meschial

**Affiliations:** I Universidade Federal do Pará. Belém, Pará, Brazil.; II Universidade do Estado de Santa Catarina. Chapecó, Santa Catarina, Brazil.; III Universidade Federal de Santa Catarina. Florianópolis, Santa Catarina, Brazil.

**Keywords:** Professional Practice Management, Health Management, Organization and administration, Nursing Care, Nursing, Gestión de la Práctica Profesional, Gestión en Salud, Organización y Administración, Atención de Enfermería, Enfermería, Gerenciamento da Prática Profissional, Gestão em Saúde, Organização e Administração, Cuidados de Enfermagem, Enfermagem

## Abstract

**Objectives:**

to clarify the concepts of Nursing Care Management and Nursing Care Administration in the scientific literature, highlighting approximations and distances between the terms.

**Methods:**

scoping review as per Joanna Briggs Institute protocol and preferred reporting items for Systematic Reviews and Meta-analysis extension for Scoping Reviews (PRISMA-ScR). The searches were performed in LILACS, CINAHL, MEDLINE, and Scopus databases.

**Results:**

the qualitative analysis, through content analysis, counted 49 studies published between 2007 and 2020. Hospital care was the most evident level of care. It was identified that nursing care management aims at the macropolitical performance of nurses and mobilizes skills essentially strategic-cognitive, while Nursing Care Administration aims at the micropolitical performance of nurses, requiring essentially strategic-administrative skills.

**Final Considerations:**

the study allowed us to propose the conceptualization of the terms and identify the approximations and distances between them.

## RESUMO

**Objetivos:**

esclarecer os conceitos de Gestão do Cuidado de Enfermagem e Gerência do Cuidado de Enfermagem na literatura científica, destacando aproximações e distanciamentos entre os termos.

**Métodos:**

revisão de escopo conforme protocolo do Joanna Briggs Institute e Itens de Relatórios Preferenciais para Revisões Sistemáticas e extensão de Meta-Análises para Revisões de Escopo (PRISMA-ScR). As buscas ocorreram nas bases LILACS, CINAHL, MEDLINE e Scopus.

**Resultados:**

a análise qualitativa, mediante a análise de conteúdo, contou com 49 estudos, publicados entre 2007 e 2020. A Atenção Hospitalar foi o nível de atenção mais evidenciado. Identificou-se que a Gestão do Cuidado de Enfermagem objetiva a atuação macropolítica do enfermeiro e mobiliza competências essencialmente estratégico-cognitivas, enquanto a Gerência do Cuidado de Enfermagem visa à atuação micropolítica do enfermeiro, requisitando competências essencialmente estratégico-administrativas.

**Considerações Finais:**

o estudo possibilitou propor a conceituação dos termos e identificar as aproximações e distanciamentos entre eles.

## RESUMEN

**Objetivos:**

aclarar conceptos de Gestión de Atención de Enfermería y Administración de Atención de Enfermería en la literatura científica, destacando aproximaciones y alejamientos entre los términos.

**Métodos:**

revisión de ámbito conforme protocolo del Joanna Briggs Institute e Items de Informes Preferenciales para Revisiones Sistemáticas y extensión de Meta-Análisis para Revisiones de Ámbito (PRISMA-ScR). Las búsquedas ocurrieron en las bases LILACS, CINAHL, MEDLINE y Scopus.

**Resultados:**

el análisis cualitativo, mediante el análisis de contenido, cuenta con 49 estudios, publicados entre 2007 y 2020. La Atención Hospitalaria fue el nivel de atención más evidenciado. Identificado que la Gestión de la Atención de Enfermería objetiva la actuación macropolítica del enfermem y moviliza competencias esencialmente estratégico-cognitivas, mientras la Administración de la Atención de Enfermería objetiva a la actuación micropolítica del enfermera, demandando competencias esencialmente estratégico-administrativas.

**Consideraciones Finales:**

el estudio posibilitó proponer la conceptuación de los términos e identificar las aproximaciones y alejamientos entre esos.

## INTRODUCTION

Nursing work is organized into five processes: assisting, administering, teaching, researching, and participating politically^(1)^. The “assisting” process is aimed at caring for individuals, families, and communities, and the “administering” process is aimed at employing material resources and mobilizing people to make the assist process effective. In both, the nurse is the regular agent, even if using different instruments and with distinct purposes over time.

It shows the intimacy between these processes, and their complementarity in nursing work, which has been highlighted when we understand the concepts of Nursing Care Management and Nursing Care Administration, no longer conceiving them as distinct, separate processes. Thus, from the moment the diffusion of these two concepts begins, an articulation between the managerial-administrative and assistance work processes emerges^(2)^.

The articulated vision of the “administrate” and “assist” processes is relevant, especially for management practices promotion centered not only on the nursing structure or team but also on the user. This view is related to Latin America, especially Brazil, which has specific regulations for the differentiation of the terms Management and Administration in health services through NOBSUS/01-96^(3)^. In English-speaking countries, ‘management’ is a usual term for both concepts. Thus, the terms Care Management and Care Administration are a national construction, the result of the development of studies on the nursing work process, aiming at the integration of these dimensions in the work of nurses^(2-4)^.

In recent years, there has been an increasing publication of studies using the terms Nursing Care Management and Administration, some even presenting concepts for levels of care or services^(2,4)^. However, a clear distinction between the terms is not yet possible since” management” and “administration” are often used synonymously in articles. This conceptual indistinction generates reflections in practice and makes translation difficult. Thus, the distinction between the concepts of Management and Administration of Nursing Care and the delineation of their characteristics and applicability are essential to clarify the doubts and gaps that involve the theme, as well as for practical application in the daily lives of nursing professionals.

In view of the volume of productions, the indistinct use of concepts in the literature and the intention to expand the practical applicability, this study proposes to carry out a scoping review to explore the available literature to identify the distinctions between Nursing Care Management and Nursing Care Administration and establish such concepts. In that regard, the question is: what are the concepts of Nursing Care Management and Nursing Care Administration found in scientific production? How are Care Management and Administration conceptualized in the nursing professional context at different levels of Health Care? What elements constitute approximations and distances between the terms?

## OBJECTIVES

To clarify the conceptualization of Nursing Care Management and Nursing Care Administration in the scientific literature, highlighting approximations and distances between the terms.

## METHODS

### Ethical aspects

As this is a review study, the present study is exempt from consideration by the Research Ethics Committee.

### Type of study

It is a scoping review. This review method was chosen because it examines evidence that may clarify questions that are not yet well defined^(5)^, such as the case with the concepts of Management and administration of Nursing Care.

### Methodological procedures

The study used the methodological strategy proposed in the protocol of the Joanna Briggs Institute (JBI), a guide for scope review^(6)^ with actions in 11 stages, developed from April/2020 to January/2021:1 - Preliminary literature review; 2 - Construction of the research protocol; 3 - A collection of information from the authors of the sample studies; 4 - Development of the title, objective and research question; 5 - Inclusion Criteria; 6 - Research Strategy; 7 - Data Collection and History; 8 - Theoretical discussion; 9 - Extraction of results; 10 - Analysis of evidence; and 11 - Presentation of results.

The preliminary review of the literature represents the Stage 1 and allowed both the confirmation of the originality of the study and the existence of significant literature for the development of the research; it was done through a refined search in the Cochrane Library before data collection. Thus, the studies found in this database did not participate in the selection of eligible studies.

Step 2 consisted of the research protocol construction, aiming at the criteria definition, transparency, and replicability of the process^(5)^. The research protocol was registered in the Open Science Framework platform under the identification DOI 10.17605/OSF.IO/F89ZA. Step 3, information collection, sought to survey the predominance of authors and journals according to theme and year of publication. Still, the JBI scope review protocol^(6)^ guides that the title, objectives, research question, and inclusion criteria are built based on the mnemonic term PCC (population, concept, context), action performed in Step 4.

Thus, the research question was formulated considering: population -nursing students and professionals; concepts – Nursing Care Management and Nursing Care Administration and studies that assumed one or both, described, analyzed or discussed them; and Research context – levels of Health Care, which selected studies related to the performance of the defined population in Primary, Secondary and/or Tertiary care.

### Collection and organization of data

Step 5, which corresponded to the inclusion criteria, included the selected studies that made up the sample, which fully responded to the population, concept, and context sought through the research question. The study used the Latin American and Caribbean Literature in Health Sciences (LILACS), Medical Literature Analysis and Retrieval System Online (MEDLINE), Cumulative Index to Nursing and Allied Health Literature (CINAHL), and Scopus Preview (Scopus) databases with different research strategies that guided step 6 - the search in the bases took place on April 25, 2020.

In the LILACS database, the research used the association of keywords and descriptors: Management OR administration AND care AND nursing, being classified the terms Management, Administration and Care in the field “title words” and Nursing in the field “Word.” On other bases, the terms “Nursing Care” (term used in quotation marks in the search) AND Management, both in the “title” field. No time frame was defined for the search in the databases, allowing the identification of studies in any period and expanding the scope of the findings.

After searching the databases, one of the researchers exported the files to the Rayyan QCRI® centralization tool, composing Step 7. This process of selection of studies took place through peer review, with the participation of two of the researchers. Initially, one of the researchers accessed the centralizer and selected one of the identical duplicate studies, excluding the others in each duplicity situation. The use of Rayyan QCRI® centralizer allowed both researchers to perform separated selection of eligible studies, generating some disagreements in the selection. Then, to resolve the variances presented in the first selection, the researchers dialogued and solved them based on the inclusion criteria, getting to the final sample of the study. This process took place from April to June 2020.

The process of systematic organization of the sample for conducting the research, analysis of the studies, and construction of the results used the Prisma extension for conducting scope reviews (PRISMA-ScR)^(7)^.

**Figure 1:**
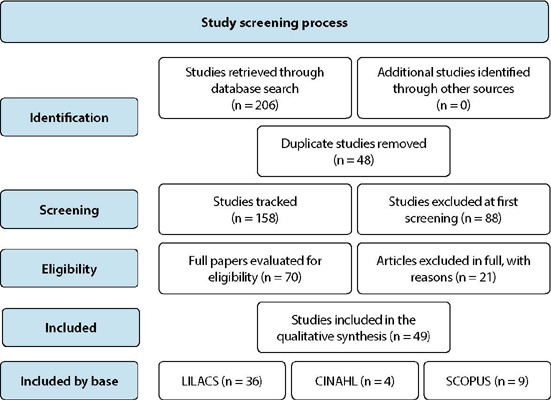
Study selection diagram - preferred reporting items for systematic diagram of systematic analyses and meta-analyses (PRISMA)

### Data analysis

The research adopted the study “The work processes in nursing^(1)^” and the NOB-SUS 01/96(3) as a conceptual framework for the fulfillment of Step 8, which includes the theoretical discussion substantiating the content analysis of the 49 studies included in the ATLAS software.it®, version 8.4.25.0, license LE_1 AB. Through this analysis using ATLAS software.it®, researchers extracted the data (title, authors, journal, country, year, level of attention, and type of study) and encrypted, attending step 9. The texts extracted from the articles were linked to what this study called as “thematic groups” to identify the approximations and distances between the concepts of Management and Administration of Nursing Care. Then, the researchers codified each thematic group again, providing the construction of approximations and distances through the analysis of these codes and their density.

This step was followed by steps 10 and 11, which consist, respectively, in the analysis of the evidence, carried out using the instrument for classifying the level of scientific evidence by type of study of the Oxford Centre for Evidence-based Medicine^(8)^; and the presentation of the results, as it follows.

## RESULTS

The study sample consisted of 49 articles (Chart 1).

**Chart 1:** Characterization of the selected studies, Belém, Pará, Brazil, 2020

Title	Country	Reference	Classification	Degree of Recommendation	Level of Evidence	Type of Study
Nursing Care Administration in hospital settings: the construction of a concept^(2)^	Brazil	CRISTOVAM, PORTO e OLIVEIRA, 2012.	Secondary care	D	5	Expert opinion devoid of critical evaluation or based on basic subjects
Conceptual analysis of Nursing Care Management in the hospital setting^(4)^	Brazil	MORORO et al., 2017.	Secondary Care	D	5	Expert opinion devoid of critical evaluation or based on basic subjects
Nurse as an integrator in the management of care for children with chronic conditions^(9)^	Brazil	MORORO et al., 2020.	Secondary Care	C	4	Case report (including cohort or lower quality case-control)
The meaning of care management for nursing teachers from the perspective of complex thinking^(10)^	Brazil	LUCCA et al., 2016.	Health care networks	C	4	A series of cases
Care management from the perspective of supervising nurses^(11)^	Brazil	COSTA et al., 2017.	Secondary Care	C	4	A series of cases
Organization of health services and management of tuberculosis care^(12)^	Brazil	BARRETO et al., 2012.	Health Care Network	C	4	A series of cases
Healthcare Management of tuberculosis: integrating a teaching hospital into primary health care^(13)^	Brazil	COELHO et al., 2016.	Health Care Network	C	4	Case report (including cohort or lower quality case-control)
Integrality and humanization in nursing care management in the Intensive Care Unit^(14)^	Brazil	MEDEIROS et al., 2016.	Tertiary Care	D	5	Expert opinion devoid of critical evaluation or based on basic subjects
Management of prenatal nursing care at a Health Center in Angola^(15)^	Brazil	SIMÃO et al., 2019.	Primary Care	C	4	A series of cases
Mental health care management from the perspective of the health care network^(16)^	Brazil	SARZANA et al., 2018.	Health Care Network	B	3B	Case-control study
Meanings of care administration constructed throughout the professional training of nurses^(17)^	Brazil	SENNA et al., 2014.	Tertiary Care	B	3B	Case-control study
Systematization of nursing care as a tool of Care Administration: a case study^(18)^	Brazil	TORRES et al., 2011.	Tertiary Care	C	4	Case-control study or poor or non-independent reference standard
Tuberculosis care management: from nurse training to practice^(19)^	Brazil	BARRETO et al., 2013.	Health Care Network	C	4	A series of cases
Management of nursing care for adolescents living with HIV/AIDS^(20)^	Brazil	KOERICH et al., 2015.	Tertiary Care	C	4	A series of cases
Organizational context and administration of care by nurses in emergency care units^(21)^	Brazil	SANTOS et al., 2014.	Secondary Care	B	3B	Case-control study
Care management actions in the Family Health Strategy^(22)^	Brazil	FERNANDES, SILVA, SILVA e MOREIRA, 2015.	Primary Care	C	4	A series of cases
Awakening new approaches to nursing care administration: a qualitative study^(23)^	Brazil	BACKES, ERDMANN, LUNARI, LUNARI FILHO e ERDMANN, 2009.	Primary Care	B	3B	Nonconsecutive selection of cases or inconsistently applied reference standard
Advanced practices for care management: reflections on the Brazilian Nursing^(24)^	Brazil	OLIVEIRA, TOSO, e MATSUDA, 2018.	Health care networks	D	5	Expert opinion devoid of critical evaluation or based on basic subjects
Care Administration and nursing governance in a maternity hospital: grounded theory^(25)^	Brazil	COPELLI et al., 2017.	Secondary Care	B	3B	Case-control study
Care management: nursing experience in a third-level care institution in the federal district^(26)^	Mexico	PONCE, CARMONA e BERNAU 2013.	Tertiary Care	D	5	Expert opinion devoid of critical evaluation or based on basic subjects
Best practices in neonatal nursing care management^(27)^	Brazil	KLOCK et al., 2019.	Tertiary Care	B	3B	Case-control study
Facilities and difficulties of nurse managers in the implementation of Care Administration in the hospital environment^(28)^	Brazil	FERNADES, da SILVA, da COSTA e de ANDRADE, 2016.	Tertiary Care	C	4	A series of cases
Nursing care management for children hospitalized with chronic conditions^(29)^	Brazil	SILVA et al., 2015.	Tertiary Care	B	3B	Case-control study
The process of dying/death: intervening conditions to the nursing care management^(30)^	Brazil	PRADO et al., 2018.	Secondary Care	B	3B	Case-control study
Approximations between social skills, Nursing Care Administration, and complex thinking^(31)^	Brazil	MONTEZELI et al., 2018.	Health care networks	D	5	Expert opinion devoid of critical evaluation or based on basic subjects
Administration of nursing care in HIV/AIDS from the palliative and hospital perspective^(32)^	Brazil	ZEPEDA et al., 2019.	Tertiary Care	C	4	A series of cases
Interactions in the management of nursing care to hospitalized children with chronic conditions: Showing intervening conditions^(33)^	Brazil	SILVA, SILVA e LEITE, 2016.	Tertiary Care	B	3B	Case-control study
Contextual aspects related to nursing care management of the child with chronic cancer pain^(34)^	Brazil	SILVA et al., 2018.	Tertiary Care	B	3B	Case-control study
Nurses’ perceptions of social skills in Care Administration from the perspective of complexity^(35)^	Brazil	MONTEZELI et al., 2018.	Tertiary Care	C	4	A series of cases
Nurse care management in the Family Health Strategy: an integrative review^(36)^	Brazil	FERNANDES E SILVA, 2013.	Primary Care	D	5	Expert opinion devoid of critical evaluation or based on basic subjects
Challenges for the administration of emergency care from the perspective of nurses^(37)^	Brazil	SANTOS et al., 2013.	Secondary Care	C	4	A series of cases
Management Of The Nursing Care In The Family Health Strategy^(38)^	Brazil	GALIZA et al., 2016.	Primary Care	C	4	A series of cases
Nursing practices in nursing and Health Care Administration: an integrative review^(39)^	Brazil	SANTOS et al., 2013.	Health care networks	D	5	Expert opinion devoid of critical evaluation or based on basic subjects
Nursing care management of clients in intensive care: content analysis^(40)^	Brazil	BARRETO, TONINI E AGUIAR, 2013.	Tertiary Care	C	4	Case series
Nursing care management for women with breast cancer in palliative chemotherapy^(41)^	Brazil	CIRILO et al., 2016.	Secondary Care	C	4	Case report (including cohort or lower quality case-control)
Care management for the hospitalized child with chronic cancer pain: intervening conditions^(42)^	Brazil	SILVA et al., 2019.	Tertiary Care	B	3B	Case-control study
The administration of care to elderly women with HIV/AIDS in an infectious-Parasitic Diseases service^(43)^	Brazil	OLIVEIRA, LEITE e FULY, 2015.	Tertiary Care	C	4	A series of cases
Factors involved in the management of nursing care: a descriptive study^(44)^	Brazil	FERNANDES, SILVA, MOREIRA, SILVA, 2013.	Primary Care	C	4	A series of cases
Improving social skills in care management provided by nurses: intervention research^(45)^	Brazil	MONTEZELI, et al., 2019.	Tertiary Care	C	4	Case report (including cohort or lower quality case-control)
Administration of nursing care in emergency care units^(46)^	Brazil	TONO DE OLIVEIRA et al., 2015.	Secondary Care	C	4	A series of cases
Nurses’ conceptions about Care Administration in an emergency department: a descriptive-exploratory study^(47)^	Brazil	SANTOS, LIMA, KLOCK e ERDMANN, 2012.	Secondary Care	C	4	A series of cases
Ethics and administration in nursing care^(48)^	Brazil	MAZUR et al., 2017.	Health care networks	D	5	Expert opinion devoid of critical evaluation or based on basic subjects
Administration of nursing care to men with cancer^(49)^	Brazil	GEFÉ DA ROSA et al., 2015.	Tertiary Care	C	4	A series of cases
Administration of nursing care through the eyes of complexity^(50)^	Brazil	BACKES, ERDMANN e MINUZZI, 2008.	Health care networks	D	5	Expert opinion devoid of critical evaluation or based on basic subjects
Nursing Care Management To Elderly Patients: The Search For Evidence^(51)^	Brazil	CHIBANTE et al., 2016.	Health care networks	D	5	Expert opinion devoid of critical evaluation or based on basic subjects
(Dis)articulations between administration and care in a surgical intensive care unit^(52)^	Brazil	BORGES e SILVA, 2013.	Tertiary Care	C	4	A series of cases
Care management: nurse leadership in the care of the person with tissue impairment^(53)^	Colombia	GONZALEZ CONSUEGRA, 2012.	Health care networks	D	5	Expert opinion devoid of critical evaluation or based on basic subjects
Communication in the management of nursing care before the death and dying process^(54)^	Brazil	PRADO et al., 2019.	Secondary Care	B	3B	Case-control study
Institutional demands and care demand in the management of nurses in an emergency room^(55)^	Brazil	MONTEZELLI, PERES E BERNARDINO, 2011.	Secondary Care	C	4	A series of cases

The study identified 139 authors, of whom 75.54% (n = 105) participated in the authorship of only one study; the other 34 participated in the authorship of two or more. It highlights the five authors with the highest number of studies conducted: Alacoque Lorenzini Erdmann (10.1%; n = 14), José Luís Guedes dos Santos (5.8%; n = 8), Joséte Luzia Leite (5.8%; n = 8), Ítalo Rodolfo Silva (4.3%; n = 6), Marcelo Costa Fernandes (3.6%; n = 5) and Aline Lima Pestana Magalhães (3.6%; n = 5).

The search found 17 journals. The one with the highest number of studies retrieved was the *Revista Brasileira de Enfermagem* with 28% (n = 12), followed by the magazine *Texto & Contexto Enfermagem* with 14% (n = 6) and the Online Brazilian Journal of Nursing and Magazine RENE, both with 11 % (n = 5). When considering the country of origin of the publications, it showed Brazil, Mexico, and Colombia, with Brazil being the country with the most expressive publication (95.92%; n = 47).

The studies that made up the sample were published between 2007 and 2020, with higher percentages in the years 2013 and 2016, both with 16.32% (n = 8), followed by 2015, 2018, and 2019 with six publications each.

The occurrence scenario presented the level of attention to which the studies corresponded. Thus, the sample articles were classified in levels of care according to the context of Health Care Networks (HCN). Among them, 36.73% (n = 18) are at the tertiary care (TC) level; 26.53% (n = 13) of the studies addressed one of the thematic groups in the context of secondary care (SC); Primary Health Care (PHC) accounted for 12.24% (n = 6) of the studies. Those who matched to more than one level of health care corresponded to the classification of HCN in its macro context, representing 24.49% (n = 12) of the studies. Thus, TC exceeded the other levels, followed by SC and HCN. PHC presented the scenario with the lowest number of publications.

When analyzing the studies by thematic group, the study found that 77.55% (n = 38) were related to the thematic group Nursing Care Administration, while 22.44% (n = 11) to the thematic group Nursing Care Management. The articles that employ the thematic core Nursing Care Administration were published in a time frame from 2007 to 2019. Regarding the frequency of studies per year, in the Nursing Care Administration group, the year that presented the highest amount was 2013, with 18.42% of the studies (n = 7), followed by 2019 with 15.7% (n = 6), 2016 and 2015 with 13.15% (n = 5) each and 2018 with 10.52% (n = 4).

On the other hand, the studies that use the Nursing Care Management Group were published in the time frame from 2012 to 2020. The year 2016 had a higher frequency of publications with this thematic group, with 27.27% of the studies (n = 3), followed by the years 2017 and 2018 with 18.18% (n = 2).

The coding of the studies generated groups of excerpts that could be classified according to their similarity. Thus, there were excerpts related to macropolitical action, in which health interventions are carried out in the expanded context of the service and the HCNs; and others to micropolitics, which the study observed the professional performance in the health service and its surroundings more often. Also, regarding the performance of professionals at the level of management and administration of nursing care, the study found competencies in the excerpts analyzed related to both groups.

In the process of coding the thematic groups, the study detected ordinary codes, varying the density.

**Table 1:** Code densities by thematic group

Code	Care Management Group	Care Administration Group
Definition	4	27
Objectives	13	40
Ways	2	15
Principles	5	24
Requirements	2	14

When analyzing the codes related to the Nursing Care Management group, the researchers noted that the concept was related to macropolitical action through excerpts of the Definition code, in which authors refer that the nurse

acts in a broad and integrative way; acts in line with public policies and under the focus of integrality; relates care management to interprofessional discussion to seek the best way to provide care [...] recognizing that managing care is inherent to the performance of nurses, in addition to evidencing the importance of communication in this process^(9)^; Meaning in a more expanded, less rigid, and more distant way from the classical model of management^(10)^

The literature shows that, for the management of nursing care, nurses use a wide range of resources^(4,9)^ - identified in the Requirements code, through the citations “ecosystem approach in the management process^(4)^,” “Leadership, interactive, communicative, cooperative relations, articulation, organization^(4, 10-12)^.”

The research observed that Care Management is focused on the proper functioning of the HCN - through the Ways Code, relating to the excerpts

Health care networks/articulation in network^(13-16)^; Build a network of integral, humanized health services [...]; they bring a systemic conception, when considering the user’s itinerary in the network and the interprofessional action for care management^(9, 12, 16)^.

In this direction, this professional must ensure adequate working conditions for the nursing team, as noted in the codes definition and form, in the citations “performed by the nurse to the nursing team, health professionals and user^(4, 11)^,” “in an integrated manner^(17)^”, “Provision of conditions for health professionals to exercise their practices^(4)^.”

The mobilization of competencies found as characteristics of Nursing Care Management was essentially strategic-cognitive, as evidenced in the Requirements code, with the following examples:

A need to understand the demands of the health-disease process^(14)^, Increasing degree of technical, scientific, affective, integrative and social demand^(4, 10-12)^, is a relevant actor for the development of practices that stimulate the capacity to produce knowledge - in action -, in order to promote the expansion of skills^(11)^, Strategic position of the nurse [...] throughout the care process^(4)^.

The performance of the nursing team and the health service is made available^(10)^, according to the Objectives code, found in the excerpts “offer a systematic and quality care”^(4, 10-11, 18)^, “To build a network of integrated health services, humanized and permeated by the process of continuing education in health and co-management”^(11-12)^. The partnership with the multi-professional team was also a characteristic observed as referring to Nursing Care Management, which, in the Characteristics code, was presented, among others, in the following excerpts: “Care Management Model”^(4, 9-12, 14, 19-20)^, “Matriculation”^(16, 19)^, “Making multi-professional partnerships is part of care management”^(14)^.

Thus, the concept presented, based on the conducted study, for the term Nursing Care Management was: Nursing Care Management is the macro-political action of nurses in the health service, for the wide use of resources, aimed at the proper functioning of the Health Care Network; it ensures, through the Systematization of Nursing Care (SNC), adequate working conditions for the nursing team, complying with professional requirements through the mobilization of essentially strategic-cognitive character - and enables the performance of other members of the nursing team, in partnership with the multidisciplinary team, ensuring the effectiveness of the citizen’s right to health through attention to the principles of the Unified Health System (SUS).

Similarly, the study proceeded to the content analysis of each code of the Nursing Care Administration thematic group. It verified the micropolitical action of nurses^(21-22)^, through the definition code, in the excerpts “the managerial attribution falls on the nurse in the health services”, “they aim to aid other people^(23)^”, “For the quality of care as a citizen’s right^(15, 17, 21-22, 24-31)^”, as well as the adoption of locoregional and network strategies, as seen in the example taken from the code Ways:

A need to work in a team in a complementary way; Interdisciplinaty^(32)^; They do not act in isolation, but with professionals from the nursing team, as well as with professionals from other teams such as the medical team^(30)^.

It also focused on the organization of his/her sector of action, selecting the following excerpts in the Ways code:

Such actions allow expanding nursing care in teamwork^(23)^; It becomes an activity carried out naturally by nurses from actions, such as the organization and coordination of the functioning of the unit^(21)^, Maintenance and control of material resources; Maintenance and control of Human capital; Personnel sizing^(2, 17, 29, 33-39)^.

The performance of the nurse, aimed at enabling nursing care and participating in it with the nursing team^(21, 28-29, 33)^, was found, among others, in the Definition and Ways codes, exemplified in the excerpts: “acts in the care and management field” “actions aimed directly or indirectly at care”^(2, 13, 18, 20, 22, 24-25, 28, 32, 34, 36, 39-44)^. On the other hand, the study observed the mobilization of competencies of an essentially strategic-administrative nature in the Requirements code, as in the quote “demands of the nurse (...) empirical knowledge and the technical and managerial skills”, also located in other studies^(2, 32, 37, 43, 45-46)^; and “it is one of the main axes of nurses’ professional performance in health services, as it understands the articulation between the care and management dimensions in the execution of their work”^(2, 43, 47)^. It also found the partnership with the multi-professional team in the Requirements code, which contextualizes the “multi-professional action through interdisciplinarity”^(13, 17, 21-22, 25, 32, 46)^.

Thus, based on the study carried out, the concept presented for the term Nursing Care Administration was: Nursing Care Administration is the micropolitical action of nurses in the health service, through the adoption of locoregional and network strategies, aimed at the organization of their sector of activity; it occurs by mobilizing essentially strategic-administrative competencies, ensured through the SNC, especially the Nursing Process (NP); and this performance enables nursing care and promotes participation to it, with the nursing team, in partnership with the multi-professional team to foster the access to integrated health care to the individual and community.

## DISCUSSION

Nursing Care Management and Nursing Care Administration are concepts whose praxis involves a theoretical-practical framework for their effectiveness. This framework is surrounded by features that both make up the structure of the action performed and direct this action to achieve its goals. The more adequately and assertively such characteristics are used, the greater the chances of success of the professional in performing such action. In this study, these characteristics were aggregated by codes generating the basis for the content analysis performed.

In the research, the authors, the journals, and the country of publication most strongly evidenced refer to Brazil. It is due to the model of care adopted in the country, which encourages the discussion of such terms distinctively, that is, with a view focused on the HCNs^(12, 13, 16, 19)^. The health care model still predominant in the country influences the levels of care with which the studies are related, developing the discussion of topics by levels as the discussion of networks evolves in the country. There is a predominance of national publications with interest focused on tertiary and secondary levels of care, however with a significant increase in studies related to linking these to other levels through the HCNs.

In this review, the term Nursing Care Administration was observed in selected publications from 2007, showing that it has been addressed for longer than Nursing Care Management, which appears for the first time among the studies collected in 2012. Nursing Care Administration has been, to date, the object of greater academic interest, with three times more studies in the composition of the sample of this research (77.55%; n = 38) than Nursing Care Management (22.44%; n = 11). It is due to the very model of health care adopted in the country. In addition, the inclusion of the term Nursing Care Management in the sample composition coincides with the period of expansion of the discussion of HCNs.

The codes found led to the conceptualization and clarification of the approximations and distances between the thematic groups through the coding of the excerpts as Definitions, Objectives, Ways, Principles, and Requirements.

### Approximations between Nursing Care Management and Administration

Some codes were common to the two thematic groups, thus generating approximations, that is, essential characteristics for both Management and Administration of Nursing Care.

Knowledge was broadly addressed in the studies^(2, 4, 10-11, 13-14, 17, 21, 26, 28, 29, 37, 42, 48-50)^, as a competence for both groups, as a requirement and attribute without which the exercise of management and administration of nursing care with excellence becomes incompatible.

Education^(10, 17, 51-52, 53)^ was also widely discussed as a requirement and suggestion for the qualification of Nursing Care Management and Administration actions. Adequate quality training focused on skills and attributes related to good practice in the management and administration of nursing care was presented as essential for these to be carried out following what is expected of nurses, as it instrumentalizes them for the potentiation and effectiveness of their activities^(20, 23, 27, 37, 39, 48)^.

The attitudes were listed as essential for the success of both contexts of action because to do it is necessary to know how to do it (have knowledge and skills) and have the conditions to act^(2, 17, 39, 48-49)^. In the list of competencies, the skills were present with the greatest representativeness. Throughout the information collection, the study confirmed the constant presence of strategic skills related to both nursing care management and nursing care administration. With this, it noticed not only that nurses must be technically prepared to perform their function, but they must also use knowledge, skills, and attitudes that strategically contribute to their adequate professional performance.

When it comes to the use of the skills required and conferred on nurses to carry out the management and administration of nursing care, the scope was also diverse, generating a need for broad and constant qualification for the professional who envisions success.

Among the skills/attitudes expected of a nurse in the provision of care, the study noticed the dedication of time and attention^(29, 41, 44, 47)^. This finding is relevant because, amid the current technological evolution, abundance of tools and shortage of time, the health service user lacks sociorelational contact(9,18, 27, 30-31, 35, 37, 48, 54). Therefore, the organization of time and planning are essential for the realization of care.

Health promotion is also seen as a general purpose as well as a social commitment of the nursing profession^(2, 4, 11, 14-17, 22, 31, 33, 35, 45, 55)^. The management and administration of nursing care are instruments with which one seeks to achieve the purposes of qualification of the nursing service through the correct execution of such functions^(10, 14, 16, 18, 20, 29, 32, 39, 47, 52)^. studies related to the Nursing Care Management group^(4, 20)^ and the Administration of Nursing Care^(25, 31, 50)^ clearly present the determination of the nurse as the one responsible for the management and administration of nursing care.

One of the most evident tools for both management and administration of nursing care was the NP^(4, 9, 17, 41, 53)^, which is considered the one that underlies the SNC^(2, 4, 39, 55)^. Therefore, the position and focus assumed by nurses in this context, evidencing their role as leaders^(4, 26, 29, 39, 50, 53)^ and in defense of comprehensive^(4, 14, 16-17, 50, 53)^ and humanized^(4, 14, 35, 36, 39, 41)^ care.

It is pertinent to highlight the relationship of the SNC and the NP with the concepts. Considering the Nursing Care Management concept presented, the relevance centered on macropolitics is understood as linked to SNC. On the other hand, for the Nursing Care Administration concept presented, micropolitics is linked to both the NCS and the NP. Thus, the SNC is expected to Care Management and Care Administration at different levels of applicability, but, by the description of the concepts, the NP is linked to Care Administration.

### Distances between Nursing Care Management and Administration

The study showed that, despite many theoretical and practical approximations between Management and Administration of Nursing Care, the objectives of Care Management are at the macro level and strategically influence the entire service/HCN. On the other hand, if we consider the Care Administration objectives, they aim to meet specific demands of the service, even if, for that, they need to communicate and involve the Health Care Network. Thus, to some extent, the conceptual direction of management and administration of NOB-96 has repercussions on the use of the expressions Management and Administration of Nursing Care^(3)^, as we will show below.

The Nursing Care Management was demonstrated as the macropolitical performance of the nurses^(4, 9, 16, 19, 54)^. Their broad, integrative performance, in line with public policies, is far from the classic management model, emphasizing the role of communication in this process — characteristics strongly presented through the Definition code in the aforementioned thematic core. Also, concerning macropolitical action, it was relevant to highlight the ecosystem focus of action and the relational skills required for action.

According to some studies^(4, 10-11, 18)^, Nursing Care Management actions mostly present objectives and intervention suggestions related to systemic change in the health service. This approach was highlighted in this thematic group, which included competencies such as network articulation and humanization to promote interprofessional action, ensuring the user’s itinerary in the network. Thus, one of the roles of the nursing care manager would be to integrally promote performance conditions for the nursing team with other professionals and health services through care, supervisory and educational practices^(11-12, 16)^.

The competencies observed in the thematic group Nursing Care Management reinforced the essentially strategic-cognitive nature of action, through which nurses develop their functions by analyzing, planning, and evaluating situations and contexts. They do so with broad actions that involve the acquired knowledge and the strategic ways of using it in a broader context, associating knowledge of the health-disease process with the ability to produce and articulate knowledge in a strategic position^(4, 9, 11-12)^.

“Permanent health education”, “co-management”, and “matrix-based strategies” are strongly adopted terms that underpin the Care Management Model^(4, 9-12, 14, 16, 19-20)^. Thus, this thematic group anchors its objectives in macroprocesses of HCN organization in a systematized, integral, humanized way, based on structural processes of the health system.

On the other hand, Nursing Care Administration was identified as the nurse’s micropolitical performance, focused on the performance in the health service^(21, 22)^, intending to provide quality assistance to citizens through interdisciplinary actions that promote the proper organization and functioning of the unit^(2, 13, 18, 20, 22, 24-25, 28, 32, 34, 36, 39-44)^. In contrast to Nursing Care Management, Nursing Care Administration, although it presents a network and uses locoregional strategies, focuses on the institution’s internal work process, improving attention and direct care to the health service user. Therefore, the administration characterizes and highlights the micropolitical performance of nurses.

The body of the studies presented principles that should guide the activities developed by nursing professionals, such as justice, competence, responsibility, and honesty^(24, 48)^. These principles are elements that contribute to achieving the objectives related to the exercise of Nursing Care Administration, considering its complexity and breadth. Competence is part of the principles listed as necessary for the Nursing Care Administration, and the list of competencies taken as necessary for it is broad and adds knowledge, skills, and attitudes^(2, 32, 37, 43, 45-46)^.

The performance of the Nursing Care Administration implies a profound moral commitment of nurses^(27)^ since the implementation of such a function is directly associated with the objectives of nursing care, among them, integrated health promotion through technical and administrative management of nursing and health teams. The conduction and viability of the work process of the nursing team are also part of their competencies: the nurse guides and participates in this managerial and care process, with the presentation of indirect and direct care actions.

Managerial functions are very relevant in the conduct of the nursing work process^(25, 31, 50)^ and are characterized as competencies of an essentially strategic-administrative nature. Among the tools that make up administrative skills, planning is perceived as the most popular^(18, 35, 38, 41)^ in addition to the highlights in this group, dimensioning, maintenance, and control of material resources as well as dimensioning of human capital.

### Study limitations

The absence of more specific descriptors may have excluded some relevant studies from the sample. In addition, the choice of works only in English, Portuguese, and Spanish may have limited the selection of more publications.

### Contributions to the field of Nursing, Health or Public Policy

This study brings relevant contributions since it obtained the answer to the research question when constructing the conceptualization of Nursing Care Management and Nursing Care Administration.

It contributes significantly to health services in general, especially in the nursing area, by supporting the direction of the nursing work process and the organization of health services as a whole, considering the importance of clarifying both concepts and highlighting the approximations and distances between them.

It also contributes to the discernment of the professional performance of nurses in both functions, thus promoting the qualification of professional practice. It responds not only to an academic and professional demand, but also to a personal question about the real conceptualization of both terms.

It is also relevant for the academic community since it contributes to teaching and research in this area, opening a range of investment possibilities related to the deepening and detailing of all that surrounds the concepts. Thus, the scope review is valuable for theoretical and conceptual advances in Nursing.

Finally, new studies can and should be carried out to investigate other aspects of the concepts presented.

## FINAL CONSIDERATIONS

The scope review carried out allowed the mapping of information of paramount relevance for the understanding of the use of the terms Nursing Care Management and Nursing Care Administration, both in the theoretical and practical field, in the scientific and professional environment.

The study identified that similar skills are required to work in Nursing Care Management and Nursing Care Administration. However, the mobilization of such abilities occurs at different frequencies and densities. Technical competencies were relevant in both thematic groups, considering them a factor of approximation, while the factors that distance them most evident were administrative and cognitive competencies.

In the thematic group Nursing Care Management, the mobilized skills have strategic-cognitive characteristics, while in the thematic group Nursing Care Administration, skills with strategic-administrative characteristics were required.

Through the exploration of the national and international literature and the analysis carried out in this study, the answer to the research question was obtained when constructing the conceptualization of Nursing Care Management and Nursing Care Administration.

It is also encouraged to carry out more studies related to the theme, aiming at deepening and expanding knowledge, given the wide range of possibilities involving the concepts studied and their surroundings.
